# Municipal solid waste management forecasting using neural networks at discharge point scale

**DOI:** 10.1038/s41598-026-38110-9

**Published:** 2026-02-02

**Authors:** Sergio De-la-Mata-Moratilla, Jose-Maria Gutierrez-Martinez, Ana Castillo-Martinez

**Affiliations:** Department of Computer Science, University of Alcala, 28801 Alcala de Henares, Spain

**Keywords:** Municipal solid waste, Prediction, Time series, Regression, Neural networks, Engineering, Environmental sciences, Environmental social sciences, Mathematics and computing

## Abstract

Urbanisation and population growth continue to accelerate waste generation, posing serious environmental and logistical challenges for the management of Municipal Solid Waste (MSW) management. The present study proposes a predictive framework for forecasting the behaviour of individual Discharge Points (DPs), with the view to enhancing decision-making in urban waste management. The necessity for localised predictions that extend beyond the scope of aggregated waste indicators is identified by research. Furthermore, it addresses the requirement for finer predictive granularity, which is capable of capturing the dynamic variations observed across DPs. The findings underscore the potential of data-driven approaches to facilitate more efficient, scalable, and intelligent waste collection planning in urban contexts by the incorporation of contextual and temporal information. By enabling accurate short-term forecasts, the proposed approach facilitates the transition from reactive to proactive collection planning, reducing operational cost and environmental footprints. Overall, the research contributes to advancing data-driven strategies for sustainable MSW management and demonstrates the potential of AI-based predictive model to support intelligent and scalable urban waste collection systems.

## Introduction

In recent decades, the global population has grown significantly, particularly in urban areas. This growth has been largely driven by migration from rural areas to urban regions motivated by the pursuit of better employment, education and living conditions. As a consequence, the accelerating pace of urbanisation has placed growing pressure on ecosystems, particularly due to the increase volume of waste being generated.

An analysis of the population and urbanization trends in 75 countries along the Belt and Road Initiative, now encompassing 155 countries^1^, reveals that 44 of these nations are currently in the acceleration phase of urbanization, while 26 have reached the terminal stage. The urban population in Belt and Road Initiative countries increased from 22% in 1950 to 49% in 2015 with projections suggesting a gradual rise by 2050^2^.

In Europe today, over 70% of the population resides in urban areas, and this proportion is projected to increase to 84% by 2050^3^. Similarly, according to World Bank Group around 4.4 billion people, currently live in cities worldwide. This number is expected to double by 2050, with almost 70% of the world’s population residing in in urban areas by that time^4^.

The environmental impacts of rapid urbanization are profound, manifesting in increased waste production, reduced biodiversity, higher CO_2_ emissions, and unsustainable resource consumption.

Regarding CO_2_ emissions, according to a study from 2017^5^, it was found that the total annual anthropogenic emissions of greenhouses gases from 2010 were 52 gigatons of equivalent CO_2_ suffering an increase of CH_4_ due to Municipal Solid Waste (MSW). Nowadays, CH_4_ generation from MSW landfills and open dumping grounds represents an 11% from which less than a 10% of this potential is currently captured and utilized. This situation joined reports that indicates urban production and consumption are estimated to account for about 70% of the global greenhouse gas emissions^6^, show the great impact MSW has over climate change and global warming and making us to rethink our wastes management approaches in the cities.

In relation to the increase of waste production, checking out a research made in 2020, there is a growing global trend in the solid waste generation representing its generation 2.01 billion tonnes per years projected to increase 70% by 2050, which means 3.40 billion tonnes annually if new measures are not taken^7^.

Consequently, cities are now at the forefront of global environmental strategies, as they represent both major sources of pollution and hubs of innovation for climate action. Governments and scientists alike are focusing on the development of smart cities, renewable energy, green infrastructure, eco-friendly urban design and low-carbon technologies as ways to address these issues, with an emphasis on reducing the ecological footprint of urban areas and promoting the circular economy as a sustainable urban model.

To achieve sustainable cities, it is needed the development of well-structured waste management strategies whose success depends on comprehensive data collection and monitoring to optimise waste systems. The use of IoT technologies and real-time data networks is essential for effective monitoring to allow improvements in waste collection and recycling processes.

Current research on MSW systems prioritise the total volume of waste collected when optimising process^8,9^. While this approach supports the development of broad resource allocation strategies, it overlooks the irregular distribution of waste across Discharge Points (DPs), highlighting the need for more precise, localised predictions to improve the overall system efficiency.

In the context of MSW management, a DP refers to an individual physical unit where waste is deposited by users, such as a container, bin or inlet in a pneumatic collection system and is monitored over time. Each DP represents the smallest operational element within the collection network capturing directly local waste disposal behaviour.

Therefore, monitoring of MSW at the DP levels offers critical insights into several aspects, such as the fill levels, enabling more targeted interventions. By improving the accuracy of predictions for each DP, cities can optimise collection routes, adjust collection frequency, allocate staff more efficiently, and strategically determine the placement of new DPs.

Although research has explored overall MSW system performance and transport routes, forecasting the specific behaviour of DPs within the network remains underdeveloped. Addressing this gap could yield more efficient and adaptive waste management strategies.

Such predictive capabilities are particularly useful for planning waste collection schedules, as they allow for the avoidance of unnecessary pick-ups and the prioritisation of critical areas. These benefits apply to both, traditional and pneumatic waste collection systems. While traditional systems can implement optimised routes more immediately, pneumatic system, despite being influenced by centralised collection triggers, can still benefit from better-informed planning and long-term design decisions. Moreover, predictive modelling can support the planning and deployment of new DPs by identifying demand patterns and usage trends. Addressing this gap provides a foundation for developing waste management strategies that are evidence-based, scalable, and better aligned with the dynamic demands of urban environment being able to reduce operational costs and improving service across traditional and pneumatic collection methods.

This study utilises data from real-world DPs within an MSW system to predict behaviour, laying a foundation for optimizing waste management. To forecast DP fill levels, different configurations of Artificial Neural Networks (ANNs) were employed aiming to find the most effective setup for improving waste collection strategies. These insights are expected to lead to enhanced waste management practices, allowing for more efficient resource allocation and improved operational decisions.

As improvement of the results and techniques found in the existing MSW forecasting literature, this study introduces a data-driven approach that advances current practice in several key aspects. First, it is adopted a finer-grained modelling perspective centred on individual DPs to enable forecasts that captures localised temporal and behavioural variability typically obscured in aggregated analyses. Second, it is used enriched real-world time series data characterised by irregular patterns, anomalies and multiple temporal resolutions reflecting realistic operational conditions. Finally, the work evaluates different ANN configurations under varying levels of aggregation providing insights into the trade-offs between model specificity and generalisation in MSW forecasting.

The structure of the paper is as follows: it begins with an overview of related research in the field, followed by a precise articulation of the project’s aims. Subsequently, a detailed account of the materials and methods employed is provided. The paper then discusses the results and concludes by presenting the main findings and their broader implications.

## Background

The related work section summarises prior research on MSW systems, underscoring the significance of forecasting individual DPs for targeted optimization—an area central to our study. The literature review establishes a robust foundation for our methodology and reaffirms the relevance of this approach.

As highlighted in the introduction, previous studies enrich our understanding of how predicting DP behaviour can enhance system performance, offering a basis for refining our methodology, particularly as MSW systems have been thoroughly analysed using various approaches across different countries^10^.

In economically challenged countries, research has explored initiatives towards circular economies in waste management, such as in Nigeria, where efforts aim to address increasing waste due to population growth and infrastructure deficits. These studies identify their weak legislation, poor funding, inadequate infrastructure, and ineffective waste handling as key obstacles to adopting a circular economy. They advocate sustainability strategies focusing on recycling, waste reduction, and material reuse to lessen dependency on raw materials and improve waste reprocessing. Although structural challenges persist, such approaches present opportunities to convert waste into energy and valuable products minimising environmental impact and promoting more efficient MSW management^11^.

Global research on MSW systems frequently assesses their environmental impacts, considering climate and environmental factors, such as rainfall and temperature, to evaluate how diverse climatic conditions and waste compositions influence environmental outcomes. These researches often includes projections of waste generation over time, along with potential leachate production, groundwater pollution, and greenhouse gas emissions^12^. Some studies further examine the advantages of bioreactor landfills, which facilitate accelerated waste decomposition through controlled recirculation of leachate and gases, offering an effective alternative to conventional landfill practices^13^.

Alternative approaches to MSW management include mathematical modelling^14^, eco-efficiency scenario analyses^15^, and IoT-enabled systems^16^, which enhance waste management at a macro level by focusing on entire systems rather than individual DPs.

A range of studies have addressed specific aspects such as waste generation prediction^17^, optimizing routing^18,19^ and improving logistical operations^20,21^. These approaches utilise diverse forecasting and classification techniques—including time series^17^, Support Vector Machine (SVM), artificial neural networks^22,23^, and deep learning^24^ to model overall system behaviour. These methodologies primarily aim to improve operational efficiency, recycling policies, and resource planning across installation or geographic areas.

Despite considerable advancements in MSW management, individual DP behaviour remains underexplored. While some papers have investigated DP distribution optimization and routing efficiency^18,19^, forecasting individual DPs has not been prioritised as a critical optimisation strategy. This gap indicates the need for a more granular approach to MSW management emphasising the forecasting of individual DPs.

Another relevant aspect identified in the literature is the time horizon considered for the forecasting. Some papers focus on long-term predictions, such as the annual evolution of the landfills often incorporating parameters like socioeconomic trends^25^ and population growth^26^ to support yearly forecasts^27^. Others concentrate on shorter horizons, predicting behaviour on a weekly basis^28^ while others still pursue short-term forecasting, reducing the time interval to just a few hours^29^.

Our study seeks to bridge this gap by developing predictive models that focus on individual DP behaviour, enabling more effective and precise optimisation strategies. By building on existing literature, this approach aims to enhance decision-making at the level of individual DPs ultimately contributing to improve system-wide efficiency.

## Objective and research questions

The objective of this study is the development of a predictive framework capable of capturing the individual behaviour of each DP as a medium for improving the efficiency of MSW management. To achieve this goal, a particular emphasis was placed on combining the full set of additional information that enriches the data of the studied time series that contains the DP filling information, rather than relying on simplified or aggregated representations containing only the time series values. To guide the study, we have formulated the following research questions (RQ):

- *RQ1:* Is it possible to obtain accurate predictions of DP filling levels combining additional fields with time series values?
- *RQ2:* Which temporal aggregation level provides the most suitable balance between noise reduction and predictive accuracy?
- *RQ3:* How does the prediction accuracy vary across different levels of structural granularity and aggregation of input data?

Developing effective forecasting models requires selecting an appropriate level of data granularity. This encompasses both temporal granularity, concerning the time resolution of the input data (such as hourly or daily intervals), and structural granularity, which defines whether predictions are made for a region, city, installation, subset of DPs or individual DPs. These choices directly affect the model’s capacity to reflect real-world waste dynamics accurately.

Assessing whether accurate predictions of DP filling levels can be improved by combining the time series values with additional fields available in the dataset, could be the key for obtaining results that really allow energy and resources savings in MSW management. Thanks to the extensive size of dataset, it facilitates the transition from reactive to proactive waste collection management. This study explored whether incorporating attributes such as those derived from timestamps, can lead to the more reliable and generalisable prediction chased. This approach may allow to adapt the forecast to the variability observed across different DPs, while maintaining reasonable complexity and computational efficiency.

## Materials and methods

For the implementation of every aspect studied along this paper, it was made use of KNIME Analytics Platform^30^, a software environment which provides modular workflows for ANN design and evaluation, ensuring transparency, flexibility and reproducibility of the experiments and the systematic comparison of the modelling strategies studied.

Therefore, to carry out the study with guarantees, a dataset containing the filling behaviour at 200 DPs over an uninterrupted four-year period was used. This data was collected from a northern Spanish city with a population of 18,000. Consequently, the original dataset comprises a total of 3.85 million records. Thanks to the size and variation of the dataset, a coherent knowledge base for analysis can be established.

Considering the filling level increment of DPs, it can be effectively modelled using time series, like approaches applied to entire waste facilities. In these time series, fill values change over intervals ranging from minutes to hours. As a result, a time series resolution at the hourly level or sub-hourly level (quarter, half or three quarters of hour) is sufficient, effectively reducing the dataset size and simplifying processing requirements^29,31^.

The following subsection details the data preparation phase required for an efficient use of this dataset, accompanied by another subsection focused on the models built and the scenarios studied.

### Data preparation

The data preprocessing stage was designed based on previous research involving traditional modelling techniques such as Linear and Lasso Regression^32^. In the present study, a combination of conventional and newly introduced methods was applied to enhance the training quality and predictive capacity of Artificial Neural Networks (ANNs) considering the different attributes of a set of time series from a dataset.

The choice of ANN model is motivated by their ability to model non-linear relationships and complex temporal patterns which are characteristic of MSW accumulation processes. This is especially relevant at the DP level where waste generation patterns are highly localised and influenced by multiple interacting factors. Unlike linear approaches, ANN can adapt to irregular behaviours, abrupt changes and heterogeneous usage patterns commonly observed in real operational data without imposing restrictive assumptions on data distribution or temporal regularity.

Regarding data, the original dataset consists of 3.85 million records, each of them reflecting an event in the filling process of the DPs, indicating not only the increase in the level of waste dumped, but also its emptying.

**Fig. 1:**
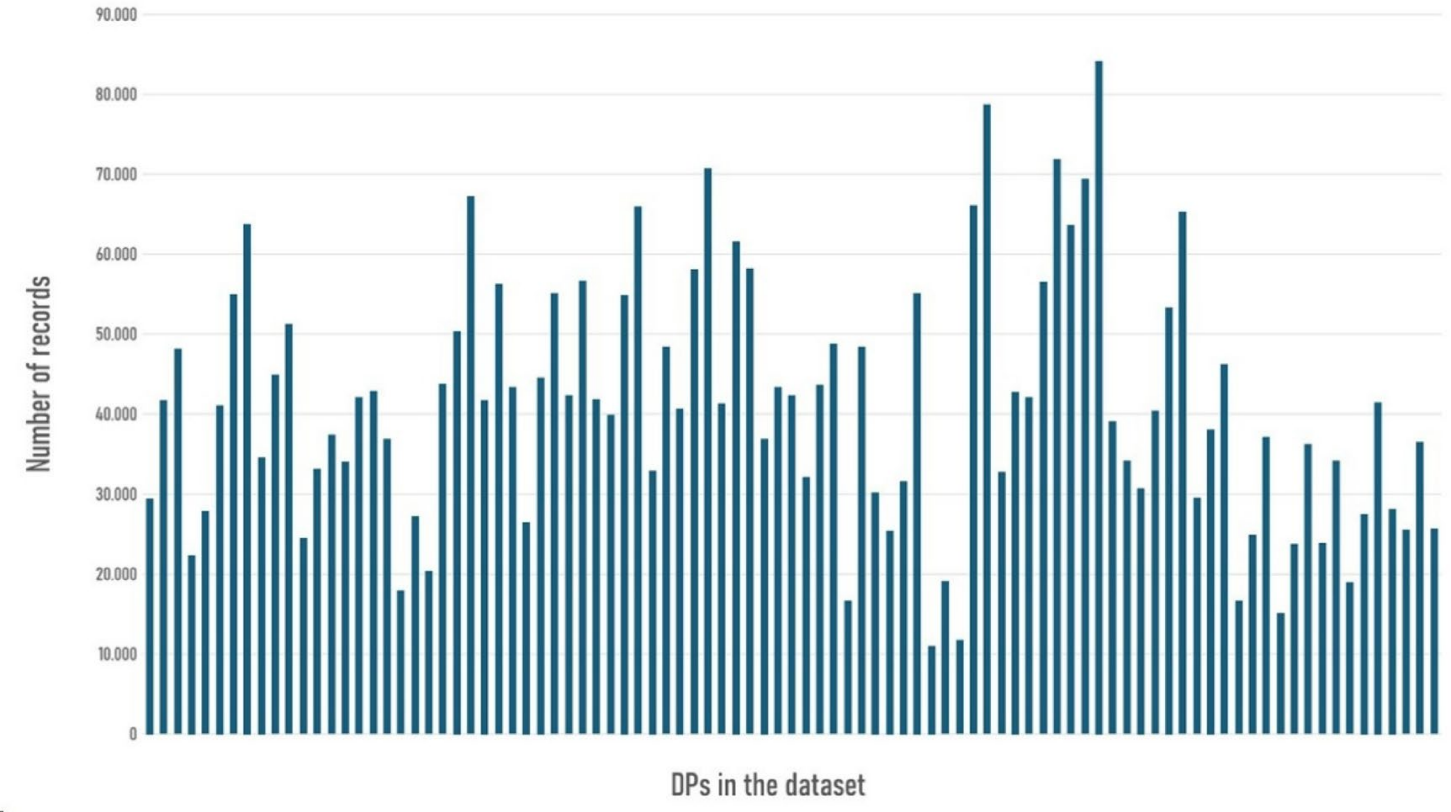
Bar diagram showing the variance in the number of records for each DP.

While the overall behaviour of the DPs could be considered homogeneous, significant differences were identified when examining several contextual factors such as their location, time of day or day of the week, have led to a variation in their number of events records. The extend of this variability was notable, while 33 DPs registered fewer than 10,000 records, with one DP containing only a single entry, two others exceeded 84,000, reaching a maximum of 84,139 records. The overall distribution showed an average of 30,535 entries per DP, a median of 33,032, and a standard deviation of 22,143. These discrepancies are illustrated in Fig. 1, which shows the differences across a representative subset of DPs.

**Fig. 2:**
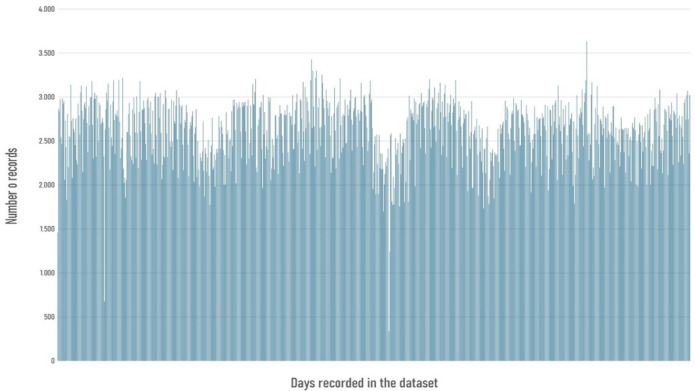
Bar diagram showing the variance in the number of records for each day from the four-year timeframe.

A comparable variability emerged when records were aggregated at the daily level. Daily totals averaged approximately 2538 entries, with a median of 2592 and a standard deviation of 363. Nonetheless, certain days registered exceptionally low levels of activities, with as few as 128 entries, while others recorded as many as 3634. This imbalance is presented in Fig. 2, which covers the entire four-year period.

Due to these differences in both DP behaviour and daily event frequencies made it necessary to implement homogenisation strategies to ensure a common structure for predictive modelling.

The first strategy consists of handling negative values, which corresponded to emptying events in the different DPs. This type of event in not considered relevant to the objective of the study as it has no impact on filling behaviour. That is why these values were recorded as zero to reflect the absence of filling during that interval.

The second strategy addressed to establish a uniform temporal resolution. As records were collected in an event-driven manner, they occurred at irregular intervals. After an examination of these temporal differences, it was observed that some events occurred in short periods of time, so it was decided to align the data in 5-min time slots to not lose relevant information, resampling the dataset with this interval. In cases where records fell outside this structure, they were adjusted to the nearest valid interval. Regarding missing values, they were generated where gaps occurred ensuring that filling increments were set to zero and incorporating additional contextual attributes such as time of day, weekday, season and holiday status. As a result of these procedures, the dataset increased to 8.74 million records.

While this higher temporal resolution improved consistency and facilitated model training providing a richer and more uniform dataset, it also introduced challenges like the finer resolution generated redundant entries during periods of inactivity, increased the level of noise, and added higher computational requirements. These limitations were acknowledged and addressed for the preparation of the data for the training of neural network models. To balance granularity and efficiency, the time resolution was reduced by aggregating the data to hourly intervals. This adjustment decreased the number of daily observations per DP from 288 to 24 and yielded a refined dataset of 3.64 million records.

Each record is composed by a combination of data related to the time series (the date and hour in which the record was obtained and the level of increment of the DP at that instant) and several additional attributes. Most of these attributes were derived from the timestamp, while others are directly associated with the DP context. The following additional attributes are available alongside the time series, enabling the search for patterns within each DP dataset: DP identifier, day of the year, day of the month, day of the week, month number, week of the month, week of the year, year, hour, minute, second, holiday indicator, season, weekday/weekend, weekend or holiday (unifying both conditions when either applies), time of day, and waste type.

While the full set was retained for this study, various subsets are typically explored to assess different input combinations, depending on the target prediction variable. In this case, as was expected as it is one of the main attributes of each time series, the variable of interest was the increment of waste, which quantifies the volume of waste deposited in a given interval. Occasionally, this value was negative due to container emptying events. These instances were recorded as zero to reflect the model’s focus on accumulation behaviour and to avoid introducing misleading signals into the learning process.

Regarding gaps in the time series often caused by temporary service interruptions due to external factors, these were filled with zero value in the increment field, assuming no change during unrecorded periods, and maintaining dataset continuity. For the remaining fields, as mentioned above, most of them were obtained according to the timestamp from the time series, therefore they were easily calculated considering that field.

To further enhance data quality, three additional techniques were introduced:

- Detection and treatment of outliers.
- Application of interpolation to restore continuity.
- Application of the normalisation of the DPs’ records.

Outlier detection was conducted using the Box and Whisker (boxplot) method, applied exclusively to the increment variable due to its volatility and critical role in forecasting. Anomalies in this field results from unexpected emptying events, equipment malfunction, or data logging errors. In contrast, other attributes being largely deterministic and less subject to abrupt variation, were excluded from this analysis. Within the dataset of 3.64 million records, a total of 185,962 were identified as anomalies.

Once anomalies were identified, they were addressed through lineal interpolation to ensure temporal coherence and preserve the overall shape of the series. This process minimised discontinuities and facilitated consistent learning across time, ensuring that the resulting data sequences were suitable for the ANN models.

Finally, predictions of DP behaviour were carried out both with and without normalisation, to determine which approach provided greater reliability under the same conditions. In the initial stage, several normalisation algorithms were tested to evaluate their compatible with different ANN configurations in the scenarios considered. The primary objectives were to minimise scale variability across records, improve models’ efficiency and explore whether this transformation revealed underlying patterns.

After these tests, it was decided to retain a single method for the subsequent stages of the study. This decision was based on the need to reduce the variability from the DPs datasets according to each of them, stabilise the models by narrowing the range of values, and concentrate on other methodological aspects more directly related to the ANN models. Among the different approaches assessed, Min–Max normalisation was selected as it consistently produced the most reliable outcomes, provided a balanced scale suitable for the learning process, and adjusted to the previously mentioned limitation.

### Preparation of study scenarios

Once the datasets were prepared, the next stage was to proceed investigating how their behaviour could be predicted under different scenarios. These scenarios were defined in terms of:

- *Structural granularity:* whether DPs were treated individually, as subsets, or as a global dataset.
- *Temporal granularity:* DPs data aggregated over intervals ranging from 1 to 6 h.
- *Pre-processing:* inclusion or exclusion of normalisation.
- *Model configuration:* number of neurons, epochs, activation functions and input features.

Regarding to the three structural granularity levels selected, they were designed with a progressive increase of size across scenarios to analyse how predictive performance evolves as local information is replaced by collective patterns. This design enables an explicit evaluation of generalisation limits in DP-level forecasting. Individual DPs represent maximum local specificity, grouped DPs capture shared behavioural patterns, and global models assess the feasibility of broad generalisation across heterogeneous units.

In relation to the different temporal aggregation levels considered in this study, they were selected to reflect realistic operational decision-making intervals in MSW collection. Sub-daily resolutions allow short-term dynamics to be captured while mitigating high-frequency noise present in the DPs data, offering a practical compromise between temporal detail and predictive stability.

With respect to normalisation, this was considered as a preprocessing step to reduce scale difference among input variables and to facilitate ANN training. Given the heterogeneous nature of the available features, including filling levels, temporal indicators and contextual variables, normalisation was evaluated to stabilise gradient updates and improve convergence behaviour. At the same time, forecasting performance was compared against the same configurations without applying normalisation.

Regarding ANN model configurations, they were selected based on a balance between representational capacity and training while sharing a common architecture across scenarios to ensure methodological consistency and comparability of results. As detailed in the following section, each ANN model consists of four layers to keep architectural complexity constant and to avoid biased performance comparison between models and scenarios. Also, two neurons’ configurations were evaluated, “200-100-50-1” and “60-100-40-1”, representing different levels of model capacity. This limited set of configurations was intentionally chosen to assess the influence of network size on predictive performance without resorting to extensive hyperparameter optimisation, thereby enhancing transparency and reproducibility.

To ensure a controlled and reliable evaluation, the analysis was restricted to a reduced set of DPs. This strategy enables the approach to be tested in a controlled setting before extending it to the full DPs dataset or to new case-specific studies in future work.

Each DP record consists of a time series complemented by additional attributes that describe the contextual conditions surrounding filling increments. These features were integrated into the predictive models studied in each scenario to enrich the input data, provide a more comprehensive basis for the DPs predictions as well as to improve the robustness of the ANN models and capture more detail about the dynamics influencing DP behaviour.

In total, three scenarios were developed as follows:

- *Scenario 1:* Individual DP predictions: Evaluates the behaviour of every DP independently by developing ANN models specifically adjusted for the characteristics of the individual DP under analysis at a given time. The approach aims to capture localised dynamics and provide models tailored to the unique patterns of each DP.
- *Scenario 2:* Grouped DPs treated as a set: In this case, selected DPs are aggregated and analysed collectively applying grouping methods such as sum, mean and median to represent the combined behaviour of the set, with the objective of identifying the patterns that emerge at the collective rather than the individual level.
- *Scenario 3:* Generic predictive behaviour: Explores the potential of a global dataset approach, where ANN models are trained across all DPs simultaneously. The goal is to generalise predictive patterns applicable to the entire system, providing insights into overarching trends that may support system-wide forecasting and decision-making.

These complementary perspectives allow to provide insights into the benefits and limitations of each modelling strategy.

Finally, it was considered the need to mitigate the risk of overfitting in the ANN models. To ensure a consistent and controlled training process across all scenarios, an early stopping strategy was applied uniformly to all configurations. If it was observed no improvement in the training loss over a patience interval of five consecutive epochs, training was terminated. This criterion was adopted to prevent unnecessary weight updates once convergence was reached, while allowing sufficient flexibility for the models to stabilise during training while this approach ensured that the differences in the predictive performance across scenarios were attributable to structural and temporal granularity choices rather than excessive model fitting.

## Results

In the following subsections, the scenarios introduced in Sect. “Materials and methods” are analysed in terms of their predictive performance. To determine the quality of the predictions, it was assessed using three metrics: Mean Absolute Error (MAE), Mean Squared Error (MSE) and coefficient of determination (R^2^). Although all three metrics are reported, R^2^ is given a particular attention as it directly reflects the alignment between observed and predicted values. While MAE and MSE provide complementary insights into absolute and squared error magnitudes, R^2^ offers an interpretable benchmark of predictive quality. In this context, although prediction with R^2^ > 0.5 might be deemed acceptable in general applications, this study adopts a more demanding threshold of 0.7, acknowledging the variability and real-time nature of the DP filling behaviour.

### Scenario 1: individual DP predictions

In this first scenario, it is explored the predictive performance of ANN models when are trained on individual DPs independently. This design aimed to achieve more accurate predictions by adapting to the unique dynamics of each DP. To enrich the time series of record, additional attributes such as holiday, season or time of day segments were incorporated.

To get a contextual idea of the forecasting performance of this scenario, a comprehensive set of 66 models, 33 trained on normalised datasets and 33 on unprocessed datasets. All models shared a four-layer architecture with an early stopping mechanism to prevent overfitting, while configurations varied in activation functions (for example ReLU and linear), number of neurons per layer, and selected input features. Along with this aspects, temporal granularity was also incorporated with records aggregated into intervals of 1, 2, 3, 4 and 6 h.

Considering the size of the results obtained from each of the situations studied for every case, the best-performing results are summarised in Tables 1 and 2, considering if the datasets are without or with normalisation respectively. Each table includes R^2^, MAE, MSE scores, as well as training and execution times.

**Table 1:** Results for Scenario 1 across the different temporal intervals without normalisation.

Hour grouping	DV	Training time (s)	Execution time (s)	R^2^	MAE	MSE
1	1	32.41	8.36	0.45	7.63	116.60
2	1	13.69	6.35	0.56	12.05	269.57
3	1	17.68	5.86	0.60	16.19	474.42
4	1	12.81	4.23	0.72	16.18	496.02
6	1	19.98	9.34	0.71	22.25	882.49
1	2	31.17	14.43	0.44	6.98	102.86
2	2	16.29	5.12	0.53	11.46	251.00
3	2	19.24	5.14	0.59	14.52	416.54
4	2	12.46	5.16	0.71	15.89	474.31
6	2	21.87	5.46	0.71	20.80	764.90
1	3	22.05	8.64	0.46	5.62	65.95
2	3	14.82	7.59	0.54	9.01	160.15
3	3	14.02	4.79	0.66	10.46	219.64
4	3	16.56	6.75	0.72	12.04	278.41
6	3	14.29	4.29	0.74	15.00	447.54
1	4	24.12	7.68	0.43	8.65	137.67
2	4	18.02	6.23	0.55	12.69	298.76
3	4	16.02	4.99	0.62	16.83	501.51
4	4	16.01	4.50	0.66	18.75	630.38
6	4	15.19	5.98	0.67	25.11	1132.59
1	5	27.17	7.26	0.46	9.00	159.18
2	5	17.99	5.48	0.57	14.22	380.82
3	5	17.55	6.57	0.63	17.54	595.47
4	5	17.30	4.52	0.69	21.58	888.65
6	5	13.02	5.32	0.69	27.85	1545.90
1	6	22.32	6.93	0.42	7.37	104.55
2	6	17.05	5.47	0.58	10.88	225.78
3	6	24.32	6.31	0.62	14.02	355.20
4	6	17.23	4.65	0.69	16.64	481.06
6	6	16.10	4.67	0.72	19.65	702.75

**Table 2:** Results for Scenario 1 across the different temporal intervals with normalisation.

Hour grouping	DV	Training time (s)	Execution time (s)	R^2^	MAE	MSE
1	70	30.14	5.78	0.39	8.50	124.81
2	70	25.07	6.15	0.36	15.42	379.07
3	70	16.88	5.06	0.43	20.12	642.72
4	70	16.97	5.20	0.67	19.24	595.54
6	70	15.10	5.47	0.68	24.20	899.98
1	89	38.72	6.66	0.38	8.01	112.26
2	89	24.16	6.23	0.40	14.13	333.89
3	89	14.99	5.60	0.45	17.74	564.40
4	89	26.49	6.76	0.66	18.61	558.38
6	89	16.30	5.09	0.67	23.53	844.97
1	101	40.02	8.33	0.36	6.61	75.52
2	101	18.02	7.44	0.40	10.60	211.30
3	101	13.93	6.47	0.48	13.13	324.21
4	101	19.67	5.37	0.67	14.18	321.64
6	101	17.07	4.97	0.62	21.05	648.26
1	134	29.54	7.37	0.30	9.93	164.57
2	134	20.55	5.41	0.38	16.10	415.45
3	134	15.68	6.60	0.44	19.62	681.94
4	134	21.18	4.93	0.56	23.73	851.71
6	134	13.33	4.57	0.60	30.16	1354.40
1	137	25.04	7.74	0.34	10.64	195.70
2	137	25.70	5.80	0.41	18.57	550.74
3	137	26.44	6.95	0.47	23.22	881.96
4	137	28.71	9.04	0.63	25.31	1059.35
6	137	15.79	4.31	0.61	35.90	1902.11
1	143	33.78	9.05	0.26	8.96	140.38
2	143	22.78	5.60	0.34	14.27	335.16
3	143	19.98	5.31	0.43	17.70	524.67
4	143	16.53	3.99	0.60	19.86	622.78
6	143	12.85	7.21	0.56	25.05	1118.12

Checking out the obtained results, it could be seen that training and execution were computationally efficient, with a total time between both remaining below one minute regardless of the temporal resolution.

Over the different ANN configurations applied, one of the most effective configurations was a four-layer ANN using the activation sequence ReLU-TanH-ReLU-Softplus. The highest-performing cases consistently involved models using the fields of day of week, hour, month, holidays and season, often complemented by the week of the month and the time of the day.

Overall, the findings suggest the first signs of promising predictions when ANNs are trained on DPs individually, particularly with 4-h and 6-h aggregations. These results highlight the potential of the approach from this scenario while also points towards the potential of exploring alternative architectures or aggregation strategies to achieve greater predictive stability and accuracy.

### Scenario 2: grouped DPs treated as a set

Following the promising results from Scenario 1, Scenario 2 investigated the predictive potential of treating the selected DPs as an aggregated group. The aim was to determine whether general predictive patterns could be captured when combining DPs with similar characteristics or behaviours.

To achieve this, three aggregation methods were applied: sum, mean and median. This approach allowed to compare the influence of the aggregation strategy on predictive performance. For each of these methods, datasets were prepared in both raw and normalised forms, producing 26 experimental cases in total each of them studying the situation for each of the 5 previously mention temporal intervals.

The results obtained are presented in Tables 3 and 4, without and applying normalisation respectively, and show the top-performing models for each temporal interval. Consistent with the previous scenario, the evaluation considered R^2^, MAE and MSE, along with training and execution times.

**Table 3:** Results for Scenario 2 across the different temporal intervals without normalisation.

Hour grouping	Type grouping	Training time (s)	Execution time (s)	R^2^	MAE	MSE
1	Sum	44.76	13.34	0.65	25.60	1247.02
1	Mean	53.90	20.94	0.68	4.13	32.16
1	Median	42.64	14.49	0.67	4.06	35.22
2	Sum	25.00	12.45	0.62	50.22	4607.56
2	Mean	60.36	11.12	0.63	8.70	130.00
2	Median	30.61	16.57	0.67	7.79	120.34
3	Sum	52.49	18.91	0.62	72.03	9304.90
3	Mean	37.31	16.66	0.76	9.60	169.63
3	Median	34.73	11.11	0.66	11.51	249.09
4	Sum	47.30	16.10	0.83	62.22	6901.15
4	Mean	49.24	12.89	0.85	9.87	172.93
4	Median	54.15	11.41	0.84	10.31	192.84
6	Sum	28.03	10.69	0.04	234.07	70,494.36
6	Mean	36.24	11.30	0.80	14.53	407.03
6	Median	34.92	9.35	0.80	14.76	425.67

**Table 4:** Results for Scenario 2 across the different temporal intervals with normalisation.

Hour grouping	Type grouping	Training time (s)	Execution time (s)	R^2^	MAE	MSE
1	Sum	92.73	19.01	0.59	29.11	1454.53
1	Mean	92.89	15.65	0.64	4.55	36.32
1	Median	103.56	17.99	0.53	5.48	49.33
2	Sum	30.79	7.23	0.59	55.60	5030.27
2	Mean	29.84	17.34	0.58	9.19	146.79
2	Median	49.62	22.08	0.57	10.01	157.94
3	Sum	27.87	11.18	0.63	72.24	9065.57
3	Mean	29.47	8.11	0.64	12.05	251.56
3	Median	27.70	9.90	0.63	12.37	269.51
4	Sum	27.40	9.48	0.77	77.28	9526.68
4	Mean	28.58	5.87	0.78	12.69	255.49
4	Median	42.08	7.83	0.77	13.49	282.11
6	Sum	22.74	6.33	0.78	91.80	16,177.90
6	Mean	43.26	15.60	0.78	17.10	452.10
6	Median	22.99	10.85	0.80	15.21	408.86

Differing from Scenario 1, training and execution costs were somewhat higher, but remained well below two minutes, confirming their feasibility in real time MSW management system contexts.

Regarding ANN architecture, it was preserved the four-layer configuration with the ReLU-TanH-ReLU-Softplus sequence proving consistently effective, although some cases highlighted the benefits of linear outputs or fully Softplus-based layers.

Regarding input features, the hour of the day proved particularly influential, often in combination with day of the week, month, holidays, season and time of day. Unlike Scenario 1, the week number of the month was less frequently decisive.

In comparison with Scenario 1, this aggregated approach led to a general improvement in predictive accuracy, though the most reliable results still achieved at broader temporal intervals (4 and 6 h), indicating the need to further refine methods for finer-grained forecasting.

### Scenario 3: generic predictive behaviour

Building on the promising outcomes of Scenarios 1 and 2, this third scenario explored whether predictive accuracy could be maintained while reducing temporal granularity. Unlike previous scenarios where DPs were either treated individually or aggregated, this approach retained the same methodological elements as the earlier scenarios, while shifting the focus to combining all the selected DPs records into a single dataset to evaluate the feasibility of developing general-purpose ANN models.

In total, 24 cases were carried out, equally divided between normalised and non-normalised datasets. As in previous scenarios, Table 5 summarised the most relevant results obtained, whether for the case was used the normalised or not datasets, presenting R^2^, MAE and MSE values together with training and execution times.

**Table 5:** Results for Scenario 3 across the different temporal intervals without and with normalisation.

Normalization	Hour grouping	Training time (s)	Execution time (s)	R^2^	MAE	MSE
No	1	161.56	36.31	0.42	7.31	105.06
Yes	1	119.31	27.15	0.41	7.22	105.29
No	2	78.95	14.05	0.51	11.18	254.24
Yes	2	59.27	24.70	0.49	11.33	263.40
No	3	41.83	13.20	0.57	14.84	413.66
Yes	3	66.54	13.52	0.55	15.53	433.75
No	4	37.89	10.93	0.65	17.31	547.69
Yes	4	22.82	11.97	0.64	17.45	552.94
No	6	29.74	10.30	0.69	21.06	821.28
Yes	6	29.17	11.95	0.68	21.56	851.10

As expected, both training and execution times increased due to the size of the unified dataset, but they remained well below the corresponding temporal intervals with less than 3 min ensuring computational feasibility.

In terms of ANN architectures, the best performing networks adopted diverse activation patterns, including ReLU–TanH–ReLU–Softplus, ReLU–TanH–ReLU–Linear and Softplus-based layers.

In terms of features, the key predictive features included time-related variables (hour, day of week, month, season, holidays and time of day), complemented by the DP identifier and, in some cases, the week number of the month.

Unlike Scenario 1 and 2, the predictive performance in this scenario was generally insufficiently for real-time application. Nevertheless, certain cases, particularly at 6-h intervals, achieved R^2^ values approaching 0.7, indicating potential for improvement. These findings suggest that further experimentation with model architectures and feature engineering could lead to more robust general-purpose forecasting models.

## Discussion

Considering the research questions and the results obtained, several insights emerge.

Regarding the RQ1, the analysis revealed clear differences between single-field and multi-field approaches. Models trained on individual features, whether from the time-series or the additional data fields, R^2^ values seldom exceeded 0.5, which is insufficient for practical application, and even the best-performing case reached only 0.68, felling short of providing a reliable forecasting tool for real time operational use. In contract, combining time series fields with additional features yielded much stronger results. Scenario 1 achieved R^2^ values up to 0.74, while Scenario 2 further improved on this performance with scores as high as 0.85. These findings highlight the necessity of leveraging richer data representations to capture the complexity of waste accumulation dynamics as well as they also align with broader evidence in time series forecasting, where feature engineering and data integration often prove critical often prove critical to accuracy.

For RQ2, the cases studied confirmed the importance of temporal aggregation in mitigating noise and revealing meaningful trends. Across the different cases of each scenario tested, aggregating data at 4- and 6-h intervals consistently improved prediction accuracy. Scenario 1 produced its strongest results with 6-h aggregation, while Scenario 2 showed flexibility at both 4- and 6-h intervals, occasionally performing better at the shorter interval. By contrast, Scenario 3 mirrored Scenario 2 general trend, but underperformed overall as was unable to exceed R^2^ of 0.7 in any configuration, suggesting limitations in this kind of setup.

For RQ3, structural granularity played a key role in predictive performance. Scenario 2 offered the most promising configuration, even without normalisation. Grouping DPs using mean or median values enhanced predictive stability, as models benefited from collective patterns rather than individual fluctuation, thereby reducing noise and increasing predictive stability. Interestingly, results without normalisation were slightly superior, suggesting that certain transformations may obscure rather than clarify the underlying patterns in the data.

Taken together, these findings highlight two practical implications. First, combining additional fields with time series features is not just beneficial, but necessary for generating forecasts of operational value. Second, when compared with previous studies, which often prioritised daily^10,33^, monthly^34,35^, or even annual^27,36^ prediction horizons, these findings suggest a shift in perspective. Sub-daily intervals, particularly 4–6 h, can provide equally or more accurate forecast striking a balance between smoothing out noise and retaining meaningful patterns, while also being computationally efficient.

From an operational standpoint, the relatively low computational requirements observed in this study suggest that these forecasting models could be integrated into real time waste collection systems without excessive hardware or processing costs. Such integration would allow municipal operators to anticipate filling patterns and optimise collection schedules according. Moreover, these findings provide a strong foundation for future research, which extend this approach to larger datasets, additional waste streams or DPs, or alternative machine learning methods.

## Conclusion

The findings of this research allow several conclusions to be drawn.

Firstly, temporal granularity played a central role in shaping predictive accuracy. Aggregation at sub-daily intervals, specifically between 1 and 6 h, generated predictions more aligned with operational contexts where decisions are required on a short-term basis. These configurations not only improved alignment with real-time tasks but also reduced the level of noise present in raw data by relying on shorter time windows, which is particularly relevant for operational decision-making in daily waste collection.

Secondly, the analysis of structural granularity showed that only the individual and grouped approaches achieved sufficiently accurate predictions for real-world application, with R^2^ values above 0.7. By contrast, the global approach failed to reach this threshold, suggesting that a one-size-fits-all model may not capture the variability of the MSW management system across the selected DPs. Therefore, further research is needed to identify models capable of improving the predictive power under a global treatment.

Thirdly, the role of normalisation proved not to be a decisive factor in this context. For individual DP predictions, omitting normalisation consistently led to better results, while for other configurations, the effects were negligible. This indicates that normalisation is not strictly required in this domain, as it does not provide a general improvement in predictive accuracy.

Moreover, enriching the models with additional variables, such as related to temporal context as time of day, seasonality or holidays, proved to be a valuable strategy, enhancing prediction accuracy and demonstrating the potential of integrating external contextual data sources to better capture the dynamics influencing the DPs behaviour.

Looking ahead, future research will aim to broaden the analysis by incorporating other contextual influences such as weather conditions such as rainfall, snow or sunshine, as well as testing new neural approaches, focusing identifying broader behavioural pattern across DPs. This will not only refine predictive accuracy in real-time applications but also expand the pool of validated models available for the deployment in operational MSW management.

Finally, the present study deliberately focused on a constrained scope in terms of the number of DPs, the range of normalisation methods and the ANN predictive models’ architectures considered, to ensure a methodological, robust and controlled evaluation. Despite these limitations, the findings highlight broader implications. The irregular nature of waste disposal, shaped by temporal, spatial, and behavioural factors, poses inherent challenges for prediction. However, by strategically selecting features, temporal intervals, and structural aggregations, much of this variability can be mitigated. Future research will therefore extend the analysis to the full DP population, explore adaptive aggregation strategies, and test and apply alternative modelling approaches to diverse urban contexts, enabling scalable and more comprehensive predictive solutions for MSW management.

## Data Availability

In this paper we have two resources that can affect to the repeatability of the experiments presented: the KNIME workflows and the dataset. The workflows developed for the research have been published via KNIME Hub, together with version information and usage references, in order to ensure full methodological transparency. The public link to access the workspace generated in KNIME Hub is: [https:/hub.knime.com/s/LnU_9pkgZMCAZW16]. It was used a dataset containing the filling behaviour at 200 DPs over an uninterrupted four-year period from a northern Spanish city with a population of 18,000. Consequently, the original dataset comprises a total of 3.85 million records. The data was acquired directly from the waste management authority responsible for the monitored area as part of routine operational data collection. Due to data ownership, confidentiality, and operational constraints, the dataset is not publicly available. In particular, it was not granted permission to disclose either the exact location of the monitored installation or the identity of the institution owning the data. The dataset may be made available for academic research purposes upon reasonable request to the corresponding author, who will forward such requests to the data owner for approval.
